# Dynamic onset response of the internal carotid artery to hypercapnia is blunted in children compared with adults

**DOI:** 10.14814/phy2.15406

**Published:** 2022-08-26

**Authors:** Christine M. Tallon, Jack S. Talbot, Kurt J. Smith, Nia Lewis, Daniela Nowak‐Flück, Mike Stembridge, Philip Ainslie, Ali M. McManus

**Affiliations:** ^1^ Centre for Heart, Lung, and Vascular Health, School of Health and Exercise Sciences University of British Columbia Kelowna British Columbia Canada; ^2^ School of Sport and Health Sciences Cardiff Metropolitan University Cardiff Wales UK; ^3^ Cerebrovascular Health, Exercise, and Environmental Research Sciences Laboratory, School of Exercise Science and Physical Health Education University of Victoria Victoria British Columbia Canada

**Keywords:** adults, cerebrovascular reactivity, children, hypercapnia, internal carotid artery

## Abstract

Intracranial blood velocity reactivity to a steady‐state hypercapnic stimulus has been shown to be similar in children and adults, but the onset response to hypercapnia is slower in the child. Given the vasodilatory effect of hypercapnia on the cerebrovasculature, assessment of vessel diameter, and blood flow are vital to fully elucidate whether the temporal hypercapnic response differs in children versus adults. Assessment of internal carotid artery (ICA) vessel diameter (ICAd), blood velocity (ICAv), volumetric blood flow (*Q*_ICA_), and shear rate (ICA_SR_) in response to a 4 min hypercapnic challenge was completed in children (*n* = 14, 8 girls; 9.8 ± 0.7 years) and adults (*n* = 17, 7 females; 24.7 ± 1.8 years). The dynamic onset responses of partial pressure of end‐tidal CO_2_ (P_ET_CO_2_), *Q*_ICA_, ICAv, and ICA_SR_ to hypercapnia were modeled, and mean response time (MRT) was computed. Following 4 min of hypercapnia, ICA reactivity and ICAd were comparable between the groups. Despite a similar MRT in P_ET_CO_2_ in children and adults, children had slower *Q*_ICA_ (children 108 ± 60 s vs. adults 66 ± 37 s; *p* = 0.023), ICAv (children 120 ± 52 s vs. adults 52 ± 31 s; *p* = 0.001), and ICA_SR_ (children 90 ± 27 s vs. adults 47 ± 36 s; *p* = 0.001) MRTs compared with adults. This is the first study to show slower hypercapnic hyperemic kinetic responses of the ICA in children. The mechanisms determining these differences and the need to consider the duration of hypercapnic exposure when assessing CVR in children should be considered in future studies.

## Graphical abstract

Differences in the temporality of cerebrovascular vasomotion between children and adults may explain previously noted distinctions in the dynamic middle cerebral artery onset response to hypercapnia. This study shows slower hypercapnic onset mean response times for internal carotid artery velocity, flow and shear rate in children compared to adults. Although dilation of the internal carotid artery was similar in children and adults after 4 minutes of hypercapnia, the onset diameter response could not be modelled.

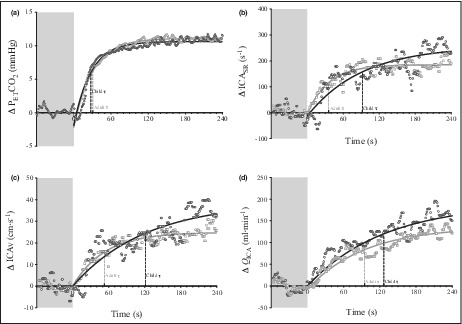

## 1 INTRODUCTION

Cerebrovascular hyperemia induced by hypercapnia is a defining trait of cerebrovascular reactivity (CVR) and provides a marker of cerebrovascular regulation (Fierstra et al., 2013; Willie et al., 2014). Evidence of developmental trajectories of CVR is limited and conflicting. In comparison with adults, gray and white matter hypercapnic CVR have been shown to be attenuated in children in response to brief bouts (45 s) of hypercapnic stimulus, suggestive of the high cerebral blood flow (CBF) in the child resulting in a reduced reserve capacity (Leung et al., 2016). In contrast, CVR estimated from the middle cerebral artery blood velocity (MCAv) response after 4 min of a step increase in inspired carbon dioxide (CO_2_) has demonstrated similar values in children and adults (Tallon et al., 2020).

The pattern of the onset response of CBF to a given stimulus can be derived from kinetic modeling and has been exploited in healthy young and older adults as well as in poststroke patients (Billinger et al., 2017; Kempf et al., 2019; Ogoh et al., 2009; Poulin et al., 1996). A single exponential model with delay term effectively describes the MCAv onset response to step increases in hypercapnia, hypoxia, and exercise, thereby providing valuable information on the speed (i.e., delay term; tau, τ and mean response time, MRT) as well as the magnitude (i.e., amplitude, Δ_A_) of the response (Billinger et al., 2017; Kempf et al., 2019; Ogoh et al., 2009; Poulin et al., 1996). The interaction between the MCAv and respiratory chemoreflex in response to hypercapnia has been explored using response kinetics in adults (Ogoh et al., 2009) and more recently compared between children and adults (Tallon et al., 2020). In children, MCAv τ was in lag to the partial pressure of end‐tidal CO_2_ (P_ET_CO_2_) τ (Tallon et al., 2020). This delay was not present in adults, confirming previous indications of developmentally distinct regulatory mechanisms in response to hypercapnia (Ellis et al., 2017). Tallon, Barker, Nowak‐Flück, Ainslie, and McManus (Tallon et al., 2020) also noted a much slower MCAv τ (~42 s slower) in children compared with adults, despite comparable increases in P_ET_CO_2_ and in MCAv amplitude. The mechanisms that account for these child–adult disparities in the onset response kinetics of the MCAv to hypercapnia are unclear, but it is likely that cerebrovascular vasomotion (changes in blood vessel diameter) plays an important role given the carotid branch arteries and intracranial arteries act as resistors to aortic outflow, protecting the cerebral microvasculature from high pulsatile energy (Zarrinkoob et al., 2016).

Adults demonstrate dilation of the internal carotid artery (ICA) in response to hypercapnia (Carr et al., 2020; Carter et al., 2016; Hoiland et al., 2017; Smith et al., 2019), and this is independent of changes in MAP and heart rate (HR) when using a transient 30 s bolus of inspired CO_2_ (Carr et al., 2020; Carter et al., 2016). Furthermore, the temporality of the responses indicates that dilation is shear‐mediated and therefore implies endothelial dependence. In older adults, shear‐mediated dilation of the ICA in response to hypercapnia is attenuated (Iwamoto et al., 2018), but the trajectory of change in ICA shear‐mediated dilation across the life span is undetermined since, to the best of our knowledge, the responsiveness of the ICA to increases in shear rate in children has yet to be assessed. Kinetic modeling of ICA velocity (ICAv), volumetric blood flow (Q_ICA_), shear rate (ICA_SR_), and diameter (ICAd) to hypercapnia provides information on the responses prior to steady state and potentially allows interrogation of the temporality of ICA vasomotion.

Therefore, the purpose of this study was to compare child hypercapnic hyperemic ICA responses and their temporal order with adult responses. We hypothesized that (i) in response to 4 min of steady‐state hypercapnia, the magnitude of the increase in ICAv, *Q*_ICA_, and ICA_SR_ would not differ between children and adults and (ii) that 4 min of sustained hypercapnia would result in similar increases in ICAd in adults and children; however, (iii) modeling of the dynamic onset response would result in a slower MRT for ICAd, Q_ICA,_ and ICA_SR_ in children compared with adults and (iv) the MRT for ICA_SR_ would precede ICAd in both children and adults.

## 2 MATERIALS AND METHODS

### 2.1 Participants

Fifty‐two participants were recruited for this study: 26 young adults (14 females; age range: 20.8 to 27.8 year) from the University of British Columbia, Okanagan campus and 26 children (15 females; age range: 8.3 to 10.9 year) from a local elementary school. Participants were eligible if they did not have any medical condition that would influence cerebrovascular or cardiopulmonary responses. The study fully conformed with the principles of the *Declaration of Helsinki* (excluding registry in a database) and was approved by the Clinical Research Ethics Board of the University of British Columbia (H16‐01281). All adult participants and parents/guardians of the children provided written informed consent. In addition, written and oral informed assent was obtained from each child.

### 2.2 Experimental protocol

Participants visited the laboratory on one occasion to complete a CVR assessment of the ICA. As per recommendations from Ainslie and Duffin (2009) participants were asked to refrain from eating high‐fat foods, consuming caffeine or alcohol, or strenuous exercise for a minimum 24 h prior to testing. Room temperature and time of day were held constant for all participants and no visual stimulation was allowed during the protocol.

Upon arrival, anthropometric measures were completed. Participants were then fully instrumented and rested supine for 10 min prior to recording baseline measures. Participants remained supine for the entirety of the protocol. Following the 10 min rest, data collection consisted of a 2 min baseline while breathing room air and 4 min of a hypercapnic stimulus, consisting of a fixed concentration of 0.06 factional inspired CO_2_ (F_I_CO_2_) in 0.21 fractional inspired oxygen (F_I_O_2_), balance nitrogen. Elevations in F_I_CO_2_ were achieved using an open‐circuit Douglas bag containing 6% CO_2_, in 21% oxygen, balance nitrogen. A three‐way Hans Rudolph valve allowed F_I_CO_2_ to be switched from room air to the Douglas bag. Measures of ICAd and ICAv were made using high‐resolution Duplex ultrasound, alongside the assessment of P_ET_CO_2_, partial pressure of end‐tidal oxygen (P_ET_O_2_), HR, and MAP.

### 2.3 Measurements

#### 2.3.1 Anthropometrics and maturation

Body mass was assessed to the nearest 0.1 kg using a beam balance scale (Detecto, USA), stature, and sitting height to the nearest 0.1 cm using a portable stadiometer (Seca Portable; Seca, Germany) barefoot and in light clothing. Body mass index (BMI, kg m^−2^) was calculated, and weight status was classified in an age‐ and sex‐specific manner using the World Health Organization standards (de Onis et al., 2007). None of the participants were obese. Of the children, one classified as thinness grade 1 and one overweight. Of the adults, four were classified as overweight. Child maturation status was assessed via two established methods: calculating predicted age at peak height velocity (aPHV) using the Mirwald equation (Mirwald et al., 2002) and parental report of Tanner stage for pubic hair and genitalia (Rasmussen et al., 2015). Briefly, Tanner stage 1, or prepubertal status, is defined by the lack of secondary sexual characteristics (i.e., no pubic hair, breast or genitalia development), and Tanner stage 2, or early pubertal status, is defined by the onset of secondary sexual characteristics (i.e., sparse pubic hair, breast buds, or onset of male genitalia growth) (Marshall & Tanner, 1969, 1970).

#### 2.3.2 Extracranial arterial measures

The participants were supine while their right ICA was assessed throughout the baseline period and 4‐min hypercapnic challenge using a 15 MHz multifrequency array vascular ultrasound (Terason T3200, Teratech, Burlington, MA). Blood velocity was assessed using pulsed‐wave mode and vessel diameter using B‐mode imaging. Test–retest reliability for ICA diameter was 1.5%. All measures of extracranial arteries followed established technical recommendations (Thomas et al., 2015). Recordings of the ICA were screen‐captured and stored as video files for offline analysis (Woodman et al., 2001). Recordings of the ICA were visually inspected before analysis and excluded if there was (i) occurrence of an overt angle change, (ii) excessive movement of the vessel as a result of high ventilation, or (iii) overall poor image quality (e.g., unclear vessel walls). Synchronized ICAv and ICAd, recordings allowed for the calculation of *Q*_ICA_ and ICA_SR_. The following equation was used to calculate *Q*_ICA_:

(1) $$\begin{array}{c} Q_{\mathrm{ICA}}\left(\mathrm{mL}\cdot \min^{-1}\right)\\ =\frac{\text{peak envelope velocity}}{2}*\left(\pi {\left(\frac{\text{diameter}}{2}\right)}^{2}\right). \end{array}$$

Calculations of ICA_SR_ were completed with the following equation:

(2) $$\begin{array}{c} \begin{array}{c} {\mathrm{ICA}}_{\mathrm{SR}}\;\left(s^{-1}\right)=\frac{\left(4*\text{peak envelope velocity}\right)}{\text{diameter}}. \end{array} \end{array}$$

All parameters of the ICA (ICAv, ICAd, *Q*_ICA_, and ICA_SR_) were down‐sampled at 1 Hz and exported into Excel for subsequent data processing.

#### 2.3.3 Cardiorespiratory measures

Beat‐by‐beat blood pressure (BP) was assessed using a Finometer Pro (Finapres Medical Systems), and HR was assessed using a three‐lead electrocardiogram (ECG; ADInstruments BioAmp ML132). Both BP and HR were sampled continuously at 1 kHz via an analog‐to‐digital converter (Powerlab, ADInstruments Colorado Springs, Colorado) and exported using LabChart at 1 Hz. A metabolic cart (Oxycon Pro, Carefusion, USA) was used to assess P_ET_CO_2_ and P_ET_O_2_. The metabolic cart was calibrated prior to each test, calibrating the volume sensor using a 3‐l syringe and gas analyzers using gases of a known concentration. Data were collected breath by breath and interpolated in second‐by‐second bins and time aligned with HR, MAP, and ICA parameters. We excluded participants if there was relative hypocapnia at baseline, defined as a baseline value of >2 SD below the child or adult mean.

### 2.4 Data processing

#### 2.4.1 Baseline and response to 4 min of hypercapnia

Baseline values were calculated from 1 min of supine rest, and steady‐state values were taken from the final 30 s of the 4‐min test. Subsequently, absolute change scores from baseline to hypercapnia (∆) were calculated for *Q*_ICA_, ICAv, ICA_SR_, and ICAd as well as for P_ET_CO_2_, P_ET_O_2_, HR, and MAP. At both baseline and hypercapnia ICA conductance (CVC) was calculated as follows:

(3) $$\begin{array}{c} \mathrm{CVC}=\frac{Q_{\mathrm{ICA}}}{\mathrm{MAP}}. \end{array}$$

Cerebrovascular reactivity in response to elevations in P_ET_CO_2_ was calculated in both absolute (CVR_Abs_) and relative (CVR_Rel_) terms:

(4) $$\begin{array}{c} \begin{array}{c} {\mathrm{CVR}}_{\mathrm{Abs}}=\frac{\text{response}\;Q_{\mathrm{ICA}}-\text{baseline}\;Q_{\mathrm{ICA}}}{\text{response}\;P_{\mathrm{ET}}{\mathrm{CO}}_{2}-\text{baseline}\;P_{\mathrm{ET}}{\mathrm{CO}}_{2}}. \end{array} \end{array}$$

(5) $$\begin{array}{c} \begin{array}{c} {\mathrm{CVR}}_{\mathrm{Rel}}=\frac{\left\{\left[\frac{\text{response}\;Q_{\mathrm{ICA}}-\text{baseline}\;Q_{\mathrm{ICA}}}{\text{baseline}\;Q_{\mathrm{ICA}}}\;\right]\times 100\right\}}{\text{response}\;P_{\mathrm{ET}}{\mathrm{CO}}_{2}-\text{baseline}\;P_{\mathrm{ET}}{\mathrm{CO}}_{2}}. \end{array} \end{array}$$

#### 2.4.2 Dynamic onset responses to hypercapnia

Prior to the analysis of the dynamic response, the ICA hemodynamic bins (1 Hz) were passed through a median filter (with a rank of 7). This filter has been detailed previously in the analysis of adult ICA responses to hypercapnia (Carter et al., 2016) and implemented in subsequent adult investigations by others (Carr et al., 2020; Hoiland et al., 2017; Iwamoto et al., 2018)

Similar to previous experiments, mono‐exponential modeling with a delay term was used to explore the onset response of P_ET_CO_2_, *Q*_ICA_, ICAv, ICA_SR_, and ICAd to hypercapnia (GraphPad Prism v.9.0.1; GraphPad Software, San Diego, CA, USA):

(6) $$\begin{array}{c} y\;\left(t\right)=y0+{∆}_{A}\left[1-e-\left(\frac{t-\mathrm{TD}}{\tau }\right)\right], \end{array}$$

where *y*(*t*) is the response at a given time; *y*_0_ is the baseline value; Δ_A_ is the baseline corrected absolute change in amplitude from baseline to asymptote; TD is the time delay, allowed to vary in order to optimize the fit; and τ is the time constant of the response (the time taken to reach 63% of the response).

The response to hypercapnia of each participant was modeled from the onset of the 6% CO_2_ stimulus (0 s). Outliers within each participants' modeled response were detected and removed to optimize the fit of the mono‐exponential model using the robust regression and outlier removal method within the GraphPad software (Motulsky & Brown, 2006). Goodness‐of‐fit (*r* ^2^ > 0.50) and normality of residuals were used to determine model acceptability. The MRT was calculated for *Q*_ICA_, ICAv, ICA_SR_, and ICAd, as:

(7) $$\begin{array}{c} \mathrm{MRT}=\mathrm{TD}+\tau . \end{array}$$

### 2.5 Statistical analysis

Data normality was checked using the Shapiro‐Wilk test (Ghasemi & Zahediasl, 2012) and subsequently verified from skewness and kurtosis for all data at baseline. Factorial ANOVAs were used to compare baseline to 4‐min hypercapnic responses by time (BL vs. hypercapnia) and age (children vs. adults). Paired and unpaired *t* tests were used to deconstruct the ANOVA main effects and interactions where necessary. CVR was compared between age groups and sex using one‐way ANOVAs. The kinetic parameters (Δ_*A*_, τ, and MRT) were compared between age groups using one‐way ANOVAs. Statistical significance was set a priori at *p ≤* 0.05. Statistical analyses were performed using SPSS (version 25, SPSS; Chicago, IL). Data are presented as mean ± SD, unless otherwise stated.

## 3 RESULTS

Data are presented for 31 of the 52 participants recruited: 14 children (6 males, 8 females) and 17 adults (10 males, 7 females). Of the 12 children who were excluded from analyses, 3 had baseline P_ET_CO_2_ > 2 SD below the child mean, 1 refused the duplex ultrasound assessment, 2 did not complete the hypercapnic challenge, and 6 were excluded as a result of poor image quality of the vessel during the hypercapnic challenge (e.g., unclear vessel walls, excessive ICA movement). Of the adults excluded, 1 was a result of <60 s of resting data, 5 had baseline P_ET_CO_2_ > 2 SD below the adult mean, and 3 as a result of poor ICA image quality.

The mean age of the children was 9.8 ± 0.7 years (8.2–10.8 years). Stature was 142.4 ± 7.6 cm, and mass was 33.9 ± 6.7 kg. Ten (5 girls, 5 boys) of the 14 children were Tanner stage 1 and 4 children (3 girls, 1 boy) were Tanner stage 2. Offset from aPHV ranged from −3.9 to −0.9 years, with a mean of −2.4 ± 1.0 years. The mean age of the 17 adults was 24.5 ± 1.8 years (20.8–27.5 years), stature was 172.3 ± 6.4 cm, and mass was 70.9 ± 10.4 kg.

### 3.1 Baseline and steady‐state response to hypercapnia

Values for all variables at baseline, during the last 30 s of the hypercapnic challenge, and the absolute delta (Δ) are presented in Table 1. At baseline, children had a significantly higher HR and lower MAP than adults. Baseline P_ET_CO_2_ was lower in children than adults. Baseline *Q*_ICA_, ICAv, ICA_SR_, and CVC were all significantly higher in children. All variables (HR, MAP, P_ET_CO_2_, P_ET_O_2_, *Q*_ICA_, ICAv, ICAd, ICA_SR_, and CVC) increased in response to hypercapnia; however, the delta response was similar for children and adults, with the exception of ΔHR, which increased significantly more in children (see Table 1).

**TABLE 1:** Baseline and hypercapnic cardiovascular, respiratory, and vascular responses in children and adults.

Parameter	Time	Children (*n* = 14)	Adults (*n* = 17)	ANOVA main effects and interaction		
				Age (*p* value)	Time (*p* value)	Age × Time (*p* value)
HR (beats min^−1^)	BL	76 ± 7	66 ± 14	**0.016**	**0.001**	**0.001**
	Hypercapnia	90 ± 8	70 ± 12			
	∆	14 ± 6	5 ± 5*			
MAP (mmHg)	BL	75 ± 9	85 ± 11	**0.018**	**0.001**	0.738
	Hypercapnia	82 ± 11	91 ± 12			
	∆	7 ± 7	6 ± 6			
P_ET_CO_2_ (mmHg)	BL	36.8 ± 2.3	39.3 ± 2.4	**0.001**	**0.001**	0.172
	Hypercapnia	46.2 ± 2.0	49.8 ± 2.1			
	∆	9.4 ± 2.3	10.4 ± 1.8			
P_ET_O_2_ (mmHg)	BL	98.1 ± 4.6	95.0 ± 5.4	**0.001**	**0.001**	0.112
	Hypercapnia	133.3 ± 4.2	126.7 ± 4.3			
	∆	35.1 ± 5.1	31.7 ± 6.3			
*Q*_ICA_ (ml min^−1^)	BL	253.8 ± 55.1	210.0 ± 41.7	**0.014**	**0.001**	0.535
	Hypercapnia	385.7 ± 65.9	327.6 ± 63.2			
	∆	131.9 ± 44.3	117.6 ± 48.4			
ICAv (cm s^−1^)	BL	59.7 ± 5.3	44.8 ± 8.5	**0.001**	**0.001**	0.142
	Hypercapnia	87.0 ± 9.4	67.0 ± 14.7			
	∆	27.3 ± 8.0	22.1 ± 10.0			
ICAd (mm)	BL	4.99 ± 0.43	5.15 ± 0.52	0.298	**0.001**	0.535
	Hypercapnia	5.09 ± 0.43	5.29 ± 0.52			
	∆	0.10 ± 0.14	0.14 ± 0.17			
ICA_SR_ (s^−1^)	BL	481.0 ± 61.3	353.9 ± 85.4	**0.001**	**0.001**	0.144
	Hypercapnia	690.0 ± 106.5	515.5 ± 139.7			
	∆	209.0 ± 72.2	161.7 ± 79.6			
CVC (mL min^−1^ mmHg^−1^)	BL	3.44 ± 0.93	2.51 ± 0.59	**0.001**	**0.001**	0.352
	Hypercapnia	4.68 ± 0.85	3.62 ± 0.69			
	∆	1.24 ± 0.42	1.11 ± 0.38			

*Note*: Values are mean ± SD. Bold text indicates *p* < 0.05.

Abbreviations: ∆, absolute change between baseline and final 30 s of hypercapnia; BL, baseline; CVC, internal carotid artery vascular conductance; HR, heart rate; ICAd, internal carotid artery diameter; ICA_SR_, internal carotid artery shear rate; ICAv, internal carotid artery velocity; MAP, mean arterial pressure; P_ET_CO_2_, partial pressure of end‐tidal carbon dioxide; P_ET_O_2_, partial pressure of end‐tidal oxygen; *Q*_ICA_, internal carotid artery blood flow.

* A significant difference between children and adults, *p* < 0.05.

**FIGURE 1:**
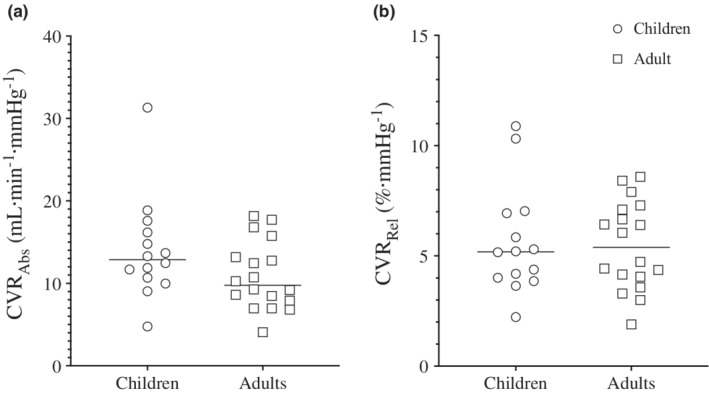
Relative and absolute cerebrovascular reactivity (CVR) of the internal carotid artery to hypercapnia by age. (a) Absolute CVR (CVR_Abs_) in children and adults. (b) Relative CVR (CVR_Rel_) in children and adults.

There were no main effects of age for CVR_Abs_ (children: 14.0 ± 7.1 ml min^−1^ mmHg^−1^ vs. adults: 11.1 ± 4.1 ml min^−1^ mmHg^−1^, *p* = 0.130; Figure 1a) or for CVR_Rel_ (children: 5.6 ± 2.5% vs. adults: 5.4 ± 2.1%, *p* = 0.792; Figure 1b).

### 3.2 Dynamic onset responses to hypercapnia

**FIGURE 2:**
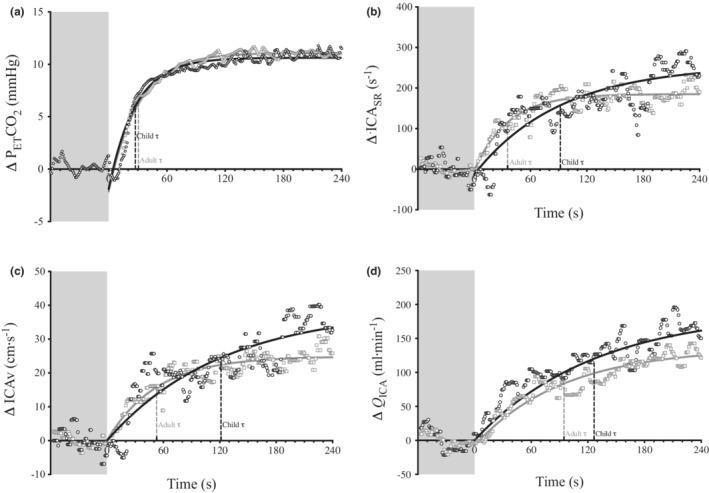
The response to hypercapnia of a representative adult and child subject. The continuous black and gray lines represent the mono‐exponential function in children and adults, respectively. The dark gray shaded area from −60 to 0 s signifies baseline; time = 0 signifies the onset of the hypercapnic stimulus.

The typical hypercapnic onset response for P_ET_CO_2_, ICA_SR_, ICAv, and *Q*_ICA_ for a representative child and adult are shown in Figure 2, and the mean response variables are provided in Table 2. The model fit was poor for ICAd, and as a result, we do not report the mono‐exponential model. The model fit for the other variables was acceptable, with the exclusion of data when *r* ^2^ < 0.5. Two children and 1 adult had *r* ^2^ < 0.5 for P_ET_CO_2_, 1 adult had *r* ^2^ < 0.5 for *Q*_ICA_, ICAv, and ICA_SR_. For these cases, a mean replacement imputation was used, resulting in an imputation of 5.5% of the data (17 of 310 data points).

**TABLE 2:** End‐tidal carbon dioxide and internal carotid artery response kinetics to hypercapnia in children and adults.

	Parameter	Children	Adults	ANOVA
		(*n* = 14)	(*n* = 17)	Age (*p* value)
P_ET_CO_2_	∆_*A*_ (mmHg)	10.5 ± 1.9	10.2 ± 1.5	0.555
	τ (s)	28.5 ± 13.5	29.8 ± 15.9	0.818
*Q*_ICA_	∆_*A*_ (ml min^−1^)	143.0 ± 70.2	120.5 ± 52.2	0.313
	τ (s)	94.7 ± 56.7	59.3 ± 37.4	**0.046**
ICAv	∆_*A*_ (cm s^−1^)	29.4 ± 7.8	21.9 ± 9.5	**0.025**
	τ (s)	101.9 ± 57.1	45.4 ± 29.7	**0.001**
ICA_SR_	∆_*A*_ (s^−1^)	205.3 ± 72.6	163.9 ± 81.3	0.149
	τ (s)	70.4 ± 21.5	40.7 ± 34.1	**0.009**

*Note*: Values are mean ± SD. Bold text indicates *p* < 0.05.

Abbreviations: ∆_A_, the change in amplitude from baseline to asymptote; ICA_SR_, internal carotid artery shear rate; ICAv, internal carotid artery velocity; P_ET_CO_2_, partial pressure of end‐tidal carbon dioxide; *Q*_ICA_, internal carotid artery blood flow; τ, the time constant of the response.

Children and adults reached a similar P_ET_CO_2_ Δ_A_, with a comparable P_ET_CO_2_ τ. There was an age difference for *Q*_ICA_ τ, with the child τ on average 35 s slower than adults. Despite a slower response in the children, the Δ_A_ for *Q*_ICA_ was similar between children and adults. Similarly, the ICAv τ was markedly slower in children; however, the ICAv Δ_A_ was greater in children compared with adults.

**FIGURE 3:**
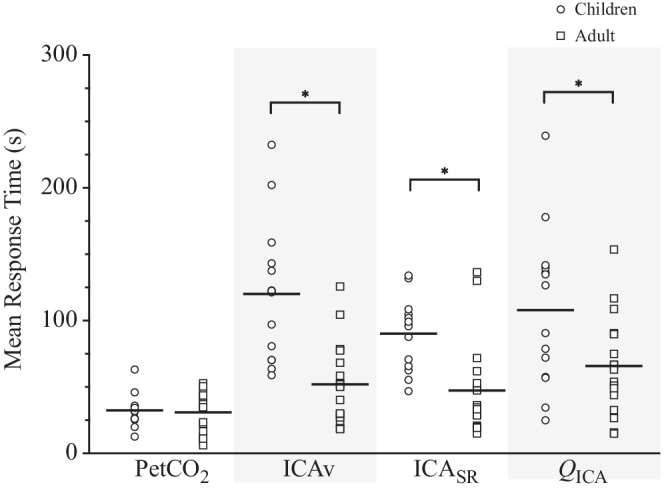
Mean response time to hypercapnia in children and adults. Symbols represent individual data. Horizontal lines are mean values. *Significant difference between children and adults, *p <* 0.05.

Figure 3 illustrates a similar MRT in children and adults for P_ET_CO_2_, but significantly slower ICAv, ICA_SR_, and *Q*_ICA_ MRT in the children compared with the adults.

## 4 DISCUSSION

This is the first study to explore ICA hemodynamic responses to hypercapnia in healthy children. We show that blood velocity, blood flow, and shear rate of the ICA increased with steady‐state hypercapnia in both children and adults and the magnitude of these responses were similar after 4 min. The ICAd also increased in response to hypercapnia in both children and adults. Although ICAd could not be modeled, the analyzed kinetics did highlight a developmental dependency on the temporal hemodynamic responses of the ICA to hypercapnia, with slower onset responses (τ and MRT) for *Q*_ICA_, ICAv, and ICA_SR_ in children. A similar ∆_*A*_ was noted for ICA_SR_ and *Q*ICA, but ∆_*A*_ ICAv was greater in children than adults.

### 4.1 Comparison of child and adult responses to steady‐state hypercapnia

Baseline measures of ICAd, ICAv, *Q*_ICA_, and ICA_SR_ in children in the present study align with those reported previously (Flück et al., 2017; Morris et al., 2017), as do the adult values (Carr et al., 2020; Smith et al., 2019). The magnitude of the hyperemic response following 4 min of steady‐state hypercapnia did not differ between children and adults. The corresponding values for CVR provide further support for this contention with no differences in absolute or relative CVR values between children and adults.

Minimal research exists pertaining to the changes in extracranial vessel diameters that occur with growth and development. In the present study, we show similar baseline ICAd in children and adults. Previous work assessing ICAd from angiograms, at a similar site in relation to the carotid bifurcation as analyzed in our video recordings, found ICAd was considerably smaller in the young children (aged 3–9 years) compared with older children and adolescents (aged 10–19 years), who had similar ICAd to the adult group (Seong et al., 2005). A limitation of the Seong, Lieber, and Wakhloo (Seong et al., 2005) work is the small sample—36 participants, with only 6 in the 3–9 years age group, 7 in the 10–19 years age group, and 14 in the adult (20–36 years) group. Additionally, the 10–19 years age group likely included individuals of widely varying maturational status. While the present study has a similar number of adults, the larger number of children within a narrower age and maturity band suggests that ICAd is no different to adult size by 8–10 years of age. A larger longitudinal study of the morphology and ensuing hemodynamic properties of the extracranial arteries is needed to confirm this. Neither Seong et al. (2005) nor this current study considered sex differences; as such, it would be imperative that hormonal changes and markers of maturation be included in future work.

The average delta diameter during steady‐state hypercapnia was small, just 2.0% in children and 2.7% in adults. These values are similar to those previously reported for the ICA (Carr et al., 2020; Hoiland et al., 2017) and were not significantly different between children and adults. Dilation of the cerebrovascular arteries is complex, given there are many factors that may influence the diameter of the vessel during a hypercapnic challenge, such as changes in cerebrovascular tone due to increases in MAP (Battisti‐Charbonney et al., 2011), which was noted in both children and adults in the current study. It is likely that, in keeping with prior findings (Hoiland et al., 2017), the sustained hypercapnic increase in MAP, as well as HR in the children, confounded the ability to discern the role shear stress plays in the regulation of ICAd. Additionally, we show an increase in P_ET_O_2_ with steady‐state hypercapnia. Although P_ET_O_2_ levels > 250 mmHg may cause a slight reduction in CBF (Willie et al., 2012), it is unlikely that the small changes in the current study would have any influence. It is clear that future studies in children will need to use alternative experimentation to hold P_ET_O_2_ constant during hypercapnia and explore alternative protocols such as the transient hypercapnic test utilized in adults (Hoiland, 2018) so that changes in P_ET_CO_2_ are not accompanied by increases in MAP and HR.

### 4.2 Comparison of child and adult ICA response kinetics to hypercapnia

Although we were unable to interrogate the temporal sequence of blood flow, shear rate, and dilation of the ICA, the response kinetics have provided unique insight into similarities and differences in the regulation of *Q*_ICA_ in response to hypercapnia across developmental stages. The *Q*_ICA_ MRT was considerably slower in children compared with young adults; however, this did not influence the magnitude of the *Q*_ICA_ response, where the amplitude was commensurate with adult values. This dissociation between the amplitude and the MRT for *Q*_ICA_ in children is intriguing. The rapid onset of response in *Q*_ICA_ with hypercapnia in adults adequately washes out the CO_2_ and resulting H+ stimulus (Hoiland et al., 2019; Ogoh et al., 2009). The high baseline *Q*_ICA_ in children may also enable a washout of CO_2_/H+ in response to the initial rise in F_I_CO_2_ without any need for a rapid increase in *Q*_ICA_. With continued exposure to increased F_I_CO_2_, *Q*_ICA_ increases, although more slowly than in adults, and it is possible this reflects regulatory processes that reduce pulsatile stress in an already highly perfused child brain (Zarrinkoob et al., 2016). Pulsatile flow in the MCA was about 50% of the PI in the carotid artery in 10‐years‐old children (Lefferts et al., 2018), and pulsatile dampening is increased in more compliant arterial beds (Zarrinkoob et al., 2016). As such, the attenuated *Q*_ICA_ onset response may reflect a greater preference for dampening pulsatile flow in the child's brain. Future investigations of cerebral pulsatility in childhood and adolescence during alterations in F_I_CO_2_ are needed and should consider the complexity of cerebral arterial physiology by considering arterial stiffness, pulse waveforms in distal and proximal cerebral arterial beds, and changes in pulsatile dampening with age, maturation, and sex (Lefferts & Smith, 2021).

Further, very little is known about the developmental changes in the mechanisms of endothelial‐dependent or ‐independent dilation of the conduit or resistance arteries. The magnitude of flow‐mediated conduit artery dilation and shear rate stimuli is related to adults but unrelated to children (Thijssen et al., 2009). Age‐dependent changes in the primary mediators of dilation of the conduit and microcirculation have been noted in isolated arteries and arterioles, evolving from prostaglandins in childhood to nitric oxide in adults (Beyer et al., 2017; Charpie et al., 1994). In adults, nonendothelial‐dependent dilation of the middle cerebral artery occurs in response to sublingual sodium nitroglycerin administration, but without increases in blood velocity, suggesting no alterations in the downstream cerebral vascular bed (Schulz et al., 2018). The influence differing vasoactive substances may have on the onset dilatatory response of the ICA in children is unknown, and pharmacologic intervention with, for example, sodium nitroglycerin or indomethacin may provide insight into the role of the prostaglandins and nitric oxide.

### 4.3 Strengths and limitations

The findings presented here are, to the best of our knowledge, the first to assess the response of the ICA to hypercapnia in children in comparison with an adult group, implementing mono‐exponential analysis to investigate the temporal response in children. It is important to acknowledge this study is not without its limitations. Twenty‐one participants were excluded from the analysis, and while this data loss was nearly 40% of the data collected, the rigorous criteria of scan acceptability and exclusion criteria allow the authors to have confidence in these preliminary findings. Weaknesses include using a fixed concentration of CO_2_, which does not provide precise targeting of the desired increase in P_ET_CO_2_ nor the ability to hold P_ET_O_2_ constant (Hoiland et al., 2019). Future investigations would benefit from using an end‐tidal forcing system or prospective gas targeting. Furthermore, bi‐lateral and regional cerebrovascular heterogeneity has not been explored. The right and left internal carotid arteries are not identical: the left is closer to the heart, arising directly from the aortic arch, whereas the right ICA originates from the brachiocephalic arch. Whether this anatomical difference induces downstream morphological and hemodynamic differences is not known. Last, we do not consider how sex differences may influence ICA hemodynamics in children or adults. There is evidence that ICA dilation increases from the low to the high estradiol phase of the menstrual cycle (Iwamoto et al., 2021) and assessment of monthly hormonal changes, although challenging, would be valuable for a more thorough developmental understanding of CBF regulation.

## 5 CONCLUSION

These novel findings broaden our insight into the hemodynamic response to hypercapnia in children. We showed a similar steady‐state ICA reactivity in children and hyperemic vasodilation. Kinetic modeling of the ICA hypercapnic onset response did not help interrogate the temporality between hemodynamic responses and dilation of the ICA but does provide insight into developmental similarities and differences in the regulation of *Q*_ICA_.

## AUTHOR CONTRIBUTIONS

Christine Tallon, Daniela Nowak‐Flück, Philip Ainslie, and Ali McManus conceived and designed the research. Christine Tallon, Nia Lewis, Daniela Nowak‐Flück, and Ali McManus assisted with data collection. Data analysis, data interpretation, or preparation of figures were completed by Christine Tallon, Jack Talbot, Kurt Smit, Mike Stembridge, and Ali McManus. Christine Tallon and Ali McManus drafted the manuscript. Christine Tallon, Jack Talbot, Kurt Smith, Nia Lewis, Daniela Nowak‐Flück, Mike Stembridge, Philip Ainslie, and Ali McManus edited, revised, and approved the final version of the manuscript.

## FUNDING INFORMATION

This work was supported by the Natural Sciences and Engineering Research Council Discovery (grant no. 201503647 to A.M.M.) and a Canadian Foundation for Innovation (to A.M.M.). KJS was also supported by an NSERC Discovery (grant no. 202006269).

## CONFLICT OF INTEREST

The authors have no conflict of interest, financial, or otherwise to declare.
